# Developed network between taxoid and phenylpropanoid pathways in *Cryptosporiopsis tarraconensis*, taxan-producing endophytic fungus by Debiased Sparse Partial Correlation (DSPC) algorithm

**DOI:** 10.1371/journal.pone.0282010

**Published:** 2023-02-23

**Authors:** Narjes Mohammadi Ballakuti, Faezeh Ghanati

**Affiliations:** Department of Plant Biology, Faculty of Biological Science, Tarbiat Modares University, Tehran, Iran; Universiti Pendidikan Sultan Idris, MALAYSIA

## Abstract

Although bioproduction of Paclitaxel by endophytic fungi is highly considered as an alternative promising source, but its yield is usually very low in comparison with other taxoids. Different strategies i.e., chemical and physical elicitations have been developed in order to overcome the shortage of Paclitaxel production. Paclitaxel biosynthesis is started with terpenoid pathway followed by phenylpropanoid metabolism where a benzoylphenylisoserine moiety is attached to C13 of baccatin III skeleton. This point which is catalyzed by the function of PAM seems to be a bottleneck that limits the rate of Paclitaxel production. Whether phenylpropanoids pathway regulates the taxanes biosynthesis in *Cryptosporiopsis tarraconensis* endophytic fungus elicited with benzoic acid (BA) was hypothesized in the present paper. The involvement of certain signal molecules and key enzymes of terpenoid and phenylpropanoid metabolism were investigated. According to the results, application of BA promoted a signaling pathway which was started with increase of H2O2 and ABA and continued by increase of NO and MJ, and finally resulted in increase of both phenylpropanoids and taxanes. However, again the rate of Paclitaxel production was lower than other taxoids, and the latter was much lower than phenolics. Therefore, supplying benzoic acid provided the precursor for the common taxan ring production. It is unlikely that Paclitaxel production is merely controlled by side chain production stage. It is more likely that in *C*. *tarraconensis* endophytic fungus, similar to Taxus sp., the competition between phenylpropanoid and taxoid pathways for substrate ended in favor of the former. The interaction network which was constructed based on DSPC algorithm confirmed that most compounds with close proximity have shared metabolic pathway relationships. Therefore, it is unlikely that the feeding with a given precursor directly result in increase of a desired metabolite which is composed of different merits.

## Introduction

The widespread demand for Paclitaxel therapy caused to search for finding of an alternative resource. Among bio productive sources of Paclitaxel, endophytic fungi are promising ones due to their faster growth rate and ease of extraction [1]. So far, about 200 endophytic fungi belonging to more than 40 fungal genera with the potential to produce taxanes have been introduced [2]. Nonetheless, none of the discovered endophytes have successfully produced taxanes on an industrial scale [2]. Different strategies e.g., application of elicitors, feeding with certain amino acids, chemical optimization of medium culture could not overcome the shortage of Paclitaxel production either [3–5]. It is likely that the development of knowledge on responsible pathways for biosynthesis of taxanes is a prerequisite for any further efforts to improve the biological production of Paclitaxel. Biosynthesis of Paclitaxel requires 2 major metabolic pathways, one providing taxan skeleton through terpenoid biosynthesis pathway and the phenylpropanoid pathways which supply the phenylisoserine side chain. The lower rate of Paclitaxel production in comparison with other taxiods may be related to the benzoylphenylisoserine side chain in C13 [6, 7]. Phenylpropanoids pathway is started with de-amination of phenylalanine to cinnamic acid catalyzed by phenylalanine ammonia-lyase (PAL). Derivatives of cinnamic acid may function as intermediates for the production of benzoylphenylisoserine Paclitaxel side chain [8].

Cinnamic acid can also be converted to benzoic acid (BA) through conjugation with coenzyme A and subsequent ẞ-oxidation [9]. It has been shown that benzoic acid not only can be used for decoration of Paclitaxel side chain but also in the structure of taxa-4,11-diene core of toxoids [10].

It has been shown that in higher plants, taxanes biosynthesis pathway is regulated by phenylpropanoid pathway and its metabolites. Benzoic acid is a phenolic acid which can be used as a substrate for biosynthesis of both cinnamic acid and taxanes structural skeleton. Such a hypothesis however, has not been tested in other taxanes producing organisms, i.e., endophytic fungi.

Switch functions of primary metabolites in the developmental regulation of certain metabolic pathways have been clarified. A few of such studies however, has been conducted considering the coverage of signal molecules, enzyme complexes, and secondary metabolites [11].

In the present research *Cryptosporiopsis tarraconensis* was used as a taxan producing endophytic fungus model to elucidate the interactions of phenylpropanoids pathways and taxiods metabolism and to clarify the mediating roles of signaling molecules e.g., H_2_O_2_, nitric oxide (NO), and hormones after elicitation with BA.

The Debiased Sparse Partial Correlation (DSPC) algorithm has been frequently applied for detection of biological networks between different compounds [12]. Metabolome analysis is a valid tool for understanding cellular networks in response to elicitors and the prediction of novel metabolic pathways [13].

## Materials and methods

### Chemicals

All chemicals and also standards of phenolic acids, flavonoids, and taxans were purchased from Sigma-Aldrich, Fluka, and ChromaDex (USA).

### Isolation, identification, growth and treatment conditions

All methods were carried out in accordance with the relevant national guidelines and legislation. According to Research Ethics Committee of Tarbiat Modares University no certificate was needed for this research. The plant samples were collected from local gardens in Iran with permissions form owners. Leaves of *Corylus avellana* were collected from different local gardens, packed on ice, and transported to the lab. After different stage of surface sterilization, they were used for isolation of endophytic fungi.

Endophytic fungus was isolated from *C*. *avellana* leaves and purified by the hyphal tip method. The purified fungus was then grown on PDB, at the darkness, 27± 2°C, on reciprocal shakers at 120 rpm. The cultures were renewed every 21 days [14]. Identification of *Cryptosporiopsis tarraconensis* was carry out using macro- and micro characteristics as described before [15]. Confirmation of identification was accomplished using ITS rDNA homology of the isolate. It was further recorded in the NCBI data base with the MW296853 accession number. After ca. 30 subcultures in PDB, BA was filter sterilized (0.25 μm) and added to 10-day old cultures at final concentrations of 0, 0.2, 1, and 1.8 mM. The different concentrations of BA were chosen based on preliminary studies as well as literatures [16]. After 11 days the mycelia were harvested using reduced pressure. Both mycelia and filtrates were frozen with liquid N2, and kept at -80°C until used for further analysis.

### H_2_O_2_, nitric oxide, and hormones

Concentration of H2O2 was quantified by the iodine method [17]. For quantitation NO, the samples were extracted with K-Pi buffer (100 mM, pH 7.0) and centrifugation (10,000×g, 15 min, 4°C). The supernatant was incubated with K-Pi (pH 7.5) and a mixture of 1% sulfanilamide and 0.1% N-1-naphthyl ethylenediamine dihydrochloride in 5% phosphoric acid solution at 27°C/ 30 min [18]. Sodium nitrite was used as standard and the absorbance of the reaction mixture was read at 540 nm [18].

For quantitation of hormones the samples were homogenized in absolute methanol (MeOH) and left overnight at 4°C. Abscisic acid (ABA), methyl jasmonate (MJ), and brassinosteroid (BR) was assessed using high-performance liquid chromatography (HPLC) (Waters, e2695, USA) following the previously described method [19].

### Enzyme activity assay

Determination of 1-deoxy-D-xylulose 5-phosphate reductoisomerase (DXR) activity was conducted using the method described by Safari et al. (2015) [20].

For measuring catalase (CAT) activity, the samples were homogenized (50 mM K-Pi buffer, pH 6.8) followed by centrifugation (12,000 ×g, 4°C). The enzyme activity was assay by monitoring the reduction of absorbance at 240 nm during one minute. For PAL and tyrosine ammonia-lyase (TAL) assay the mycelia were homogenized in Tris–HCl buffer (50 mM, pH 8.2) containing 15 mM 2-mercaptoethanol and centrifuged at 15,000 ×g, 4°C, 20min. The reaction was started by adding either 10 mM L-phenylalanine (for PAL) or 10 mM tyrosine (for TAL assay) and was paused in 100 μL 5 M HCl. EtOAc was added to mixture (3 times, 5 mL each) and evaporated. The residue was re-dissolved in MeOH. Cinnamic acid (the product of PAL) was determined at 290 nm and p-coumaric acid (the product of TAL) was detected at 330 nm (TAL) [18].

### Secondary metabolites

Extraction and quantification of taxanes were conducted by the method previously described [15].

For extraction and analysis of phenolic acids and flavonoids the samples were homogenized in MeOH containing 1% AcOH and were incubated overnight at room temperature. The supernatant was separated by centrifugation (10,000×g, 15 min, 4°C), dried, and re-dissolved in MeOH before applying for HPLC analysis. Phenolic acids were eluted by a gradient (5%-100%) MeOH: Water (consists of 2% AcOH) with a flow rate of 1 mL min-1, at 287 and 300 nm.

The flavonoids were eluted with a linear gradient (18%-82%) of acetonitrile (MeCN): distilled water (containing 0.5% O-phosphoric acid), with a rate flow of 0.8 mL min-1, and detected at 280 and 350 nm [18].

### Statistical analysis

All observations and experiments were repeated at least 3 times, each with 3 samples. Graph pad software version 5.1 was used for the variance analysis. Duncan’s new multiple-range test was used (p ≤ 0.05). Principal component analysis (PCA), hierarchical cluster analysis (HCA) and DSPC were performed by MetaboAnalyst (https://www.metaboanalyst.ca).

## Results

### Effect of BA on growth and signaling molecules

**Fig 1:**
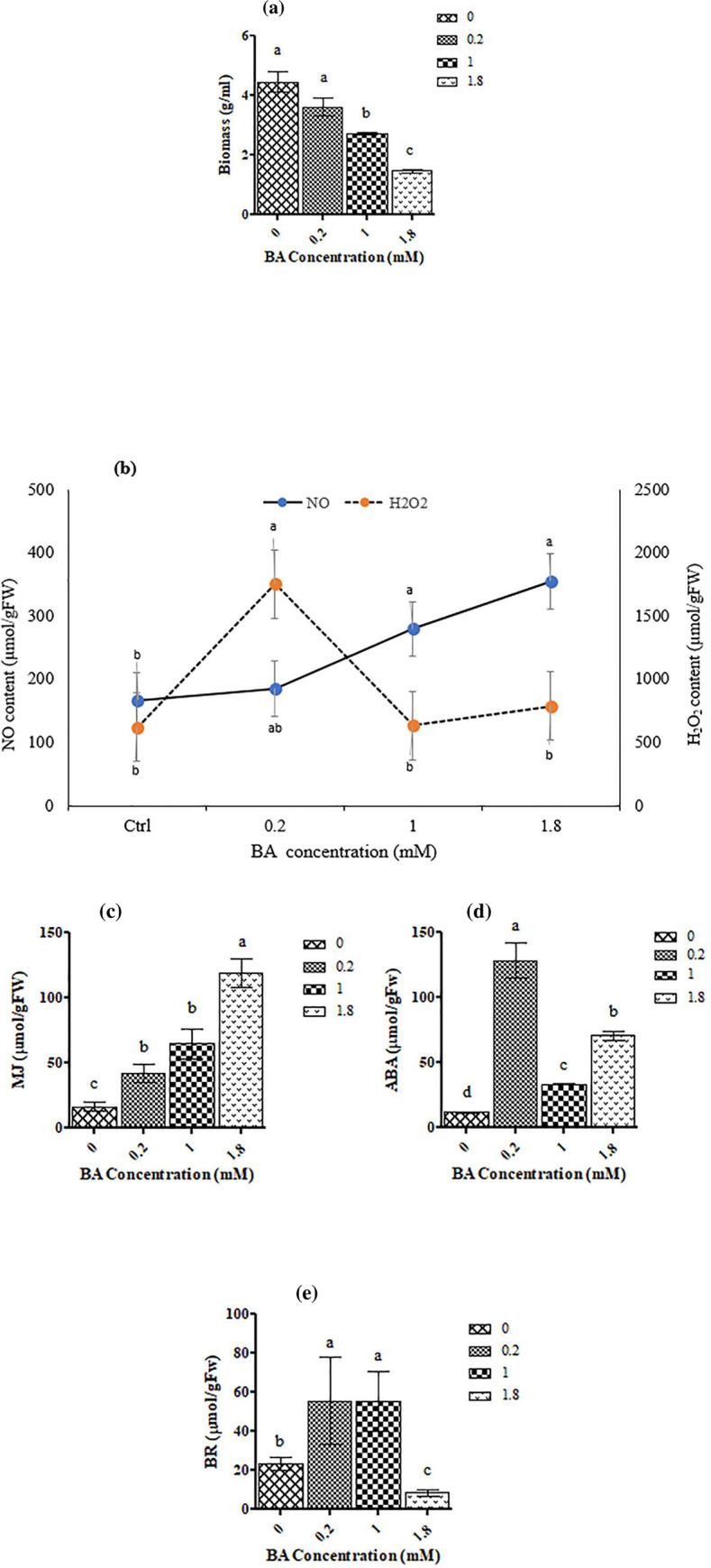
Effect of different concentrations of BA on biomass. **(a)** and signaling molecules including NO and H_2_O_2_ **(b)**, MJ **(c)**, ABA **(d)** and BR **(e)** in *C*. *tarraconensis*. Data are presented as mean ± SD, n = 3. Different letters show significant differences at *P* < 0.05 according to Duncan test.

Treatment with 0.2 mM BA had no significant effect on the growth of *C*. *tarraconensis*. At higher concentrations however, BA significantly reduced the biomass of the fungi, compared to the control (Fig 1A).

The effects of BA on the generation of signaling molecules were shown in Fig 1B–1E. As shown, 0.2 mM BA remarkably increased the H_2_O_2_ content of the fungus, while other concentrations showed no significant changes on it, compared to the controls (Fig 1B). Increasing BA supply also resulted in a more or less linear increase in NO content. The maximum content of NO was detected in those fungi which were treated with 1.8 mM BA (Fig 1B).

A similar trend was detected in concentration of MJ of BA-treated fungi, compared to the controls so that the most remarkable increase of MJ (ca. 12× of control) was recorded at 1.8 mM BA (Fig 1C).

In comparison with the control group, treatment with BA significantly increased the ABA content of the fungi (Fig 1D). However, the highest ABA content (360% of the control) was observed at 0.2 mM BA and higher concentrations of it reduced the content of ABA (Fig 1D).

Exposure of *C*. *tarraconensis* to 0.2- and 1-mM BA remarkably increased the content of BR of the fungi (ca. 2.4-fold of the control) (Fig 1E). The supply of BA at 1.8 mM however reduced the BR level of the fungus to half of the control (Fig 1E).

Principal component analysis showed a positive correlation between NO and MJ content (r = 0.81), and among H_2_O_2_ with ABA and BR content (r = 0.86, r = 0.67, respectively).

### Secondary metabolites and involved enzymes

**Fig 2:**
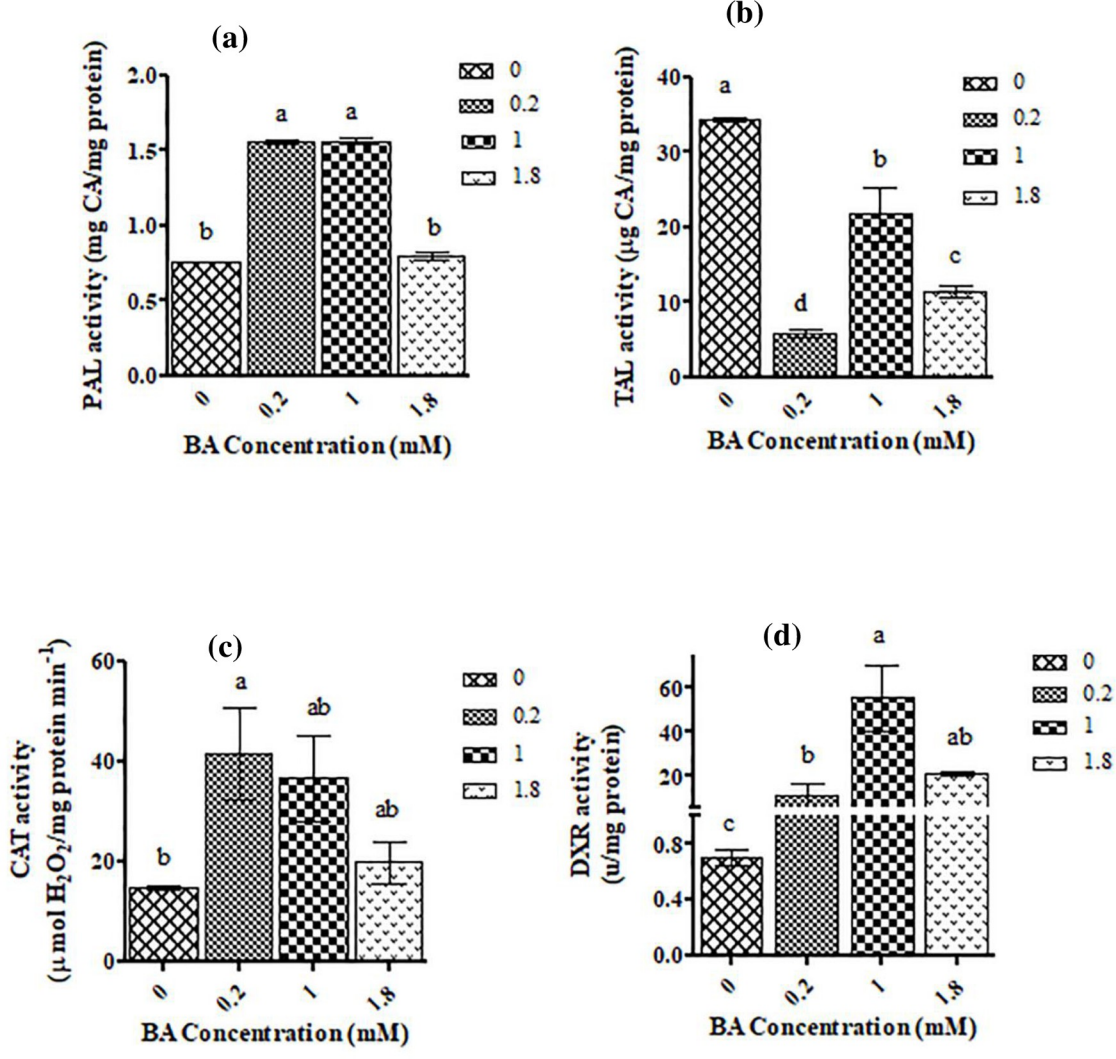
The activity of key enzymes PAL. **(a),** TAL **(b),** CAT **(c)** and DXR **(d)** involved in signaling molecules, phenolics, and terpenoids metabolism in *C*. *tarraconensis* treated with different concentrations of BA. Data are presented as mean ± SD, n = 3. Different letters show significant differences at *P* < 0.05 according to Duncan test.

Application of 0.2- and 1-mM BA increased the activity of PAL up to 2-fold of the control (Fig 2A). At 1.8 mM BA, the activity of PAL in treated samples was identical to the control (Fig 2A).

The activity of TAL, in all concentrations of BA was significantly lower than the control (Fig 2B). The activity of CAT significantly increased by 0.2 mM of BA, but at other concentrations did not show significant differences with control (Fig 2C).

The activity of DXR as a major enzyme in the terpenoid biosynthesis pathway, increased by BA treatment outstandingly (up to ×70 of the control) (Fig 2D).

**Fig 3:**
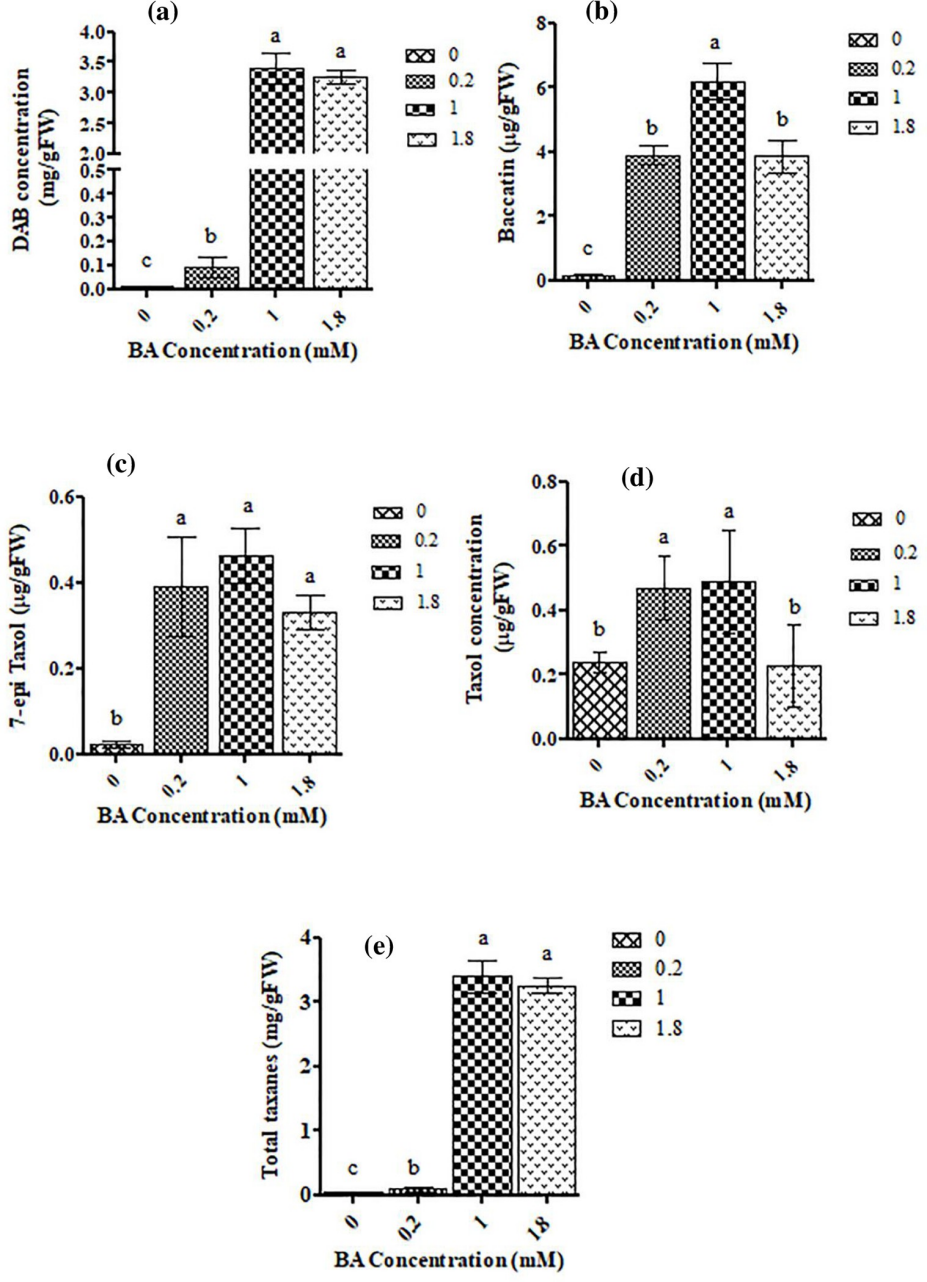
The content of DAB **(a)**, Baccatin **(b)**, 7- epi Paclitaxel **(c)** and Paclitaxel **(d)** in *C*. *tarraconensis* treated with various BA supply. Data are presented as mean ± SD, n = 3. Different letters show significant differences at P < 0.05 according to Duncan test.

Taxan profile of *C*. *tarraconensis* showed that BA treatment drastically induced the production of DAB (10-deacetyl baccatin III), compared to control. The highest amounts of DAB were quantified at 1- and 1.8-mM BA (about 300-fold of control) (Fig 3A). The content of Baccatin III also increased remarkably by BA (up to 41-fold of the control), however, showed no significant differences among different BA concentrations (Fig 3B). The taxan profile of *C*. *tarraconensis* also showed an almost 23-fold increase in the quantity of 7-epi Taxol, compared to the control (Fig 3C). Paclitaxel production also significantly increased by 0.2 mM and 1 mM BA (2-fold of control), but at 1.8 mM BA showed no significant differences from the control (Fig 3D). In comparison with control, in all applied concentrations of BA total content of toxoids was remarkably high and the highest taxoid content was detected at 1 mM BA (Fig 3E).

Phenolic acids i.e., cinnamic acid, *p*-coumaric acid, caffeic acid, gallic acid, and hydroxybenzoic acid were detected and measured in *C*. *tarraconensis* (Table 1). BA treatment remarkably induced the production of cinnamic acid up to 14-fold of the control. The content of *p*-coumaric acid at 0.2 mM BA was identical to the control but very sharply increased at 1 mM and to a lower degree at 1.8 mM BA (Table 1). Caffeic acid content decreased by BA and the most decline (3.2 -fold) was recorded in 1.8 mM of BA treatment, compared to the control (Table 1). Also, fluctuations were found in other main phenolic acids i.e., gallic acid and hydroxybenzoic acid where the former increased at that increased at 0.2 mM and 1.8 mM BA and the latter increased only at 1 mM BA, compared to the controls (Table 1) treatment respectively. Compared to the control, in all BA treatments total content of phenolic acids was remarkably high. The highest phenolic acid content was detected at 1 mM BA (Table 1).

**Table 1:** Alteration of *C*. *tarraconensis*. phenolic acids content in response to various concentration of BA. Data show mean ± SD, n = 3. Different letters show significant differences at *P* ≤ 0.05 according to Duncan test.

	Cinnamic acid		*p*- Coumaric acid	Caffeic acid	Gallic acid	Hydroxybenzoic acid	Total
BA supply (mM)		(μg. g FW^-1^)					
0	0.2± 0.0^b^		0.0±0.0^c^	23±2^a^	44±3^c^	137±2 ^b^	204±7^c^
0.2	3±1.1^a^		0.1±0.0^c^	9±7^c^	382±69 ^a^	22±3 ^d^	416±80.1^b^
1.0	3±1.5^a^		550±57 ^a^	14±0.8^b^	33±4.5^d^	455±33^a^	1000±97^a^
1.8	2±1.1^a^		0.5±0.1^b^	7±6^c^	162±14 ^b^	54±9 ^c^	225±30^c^

**Fig 4:**
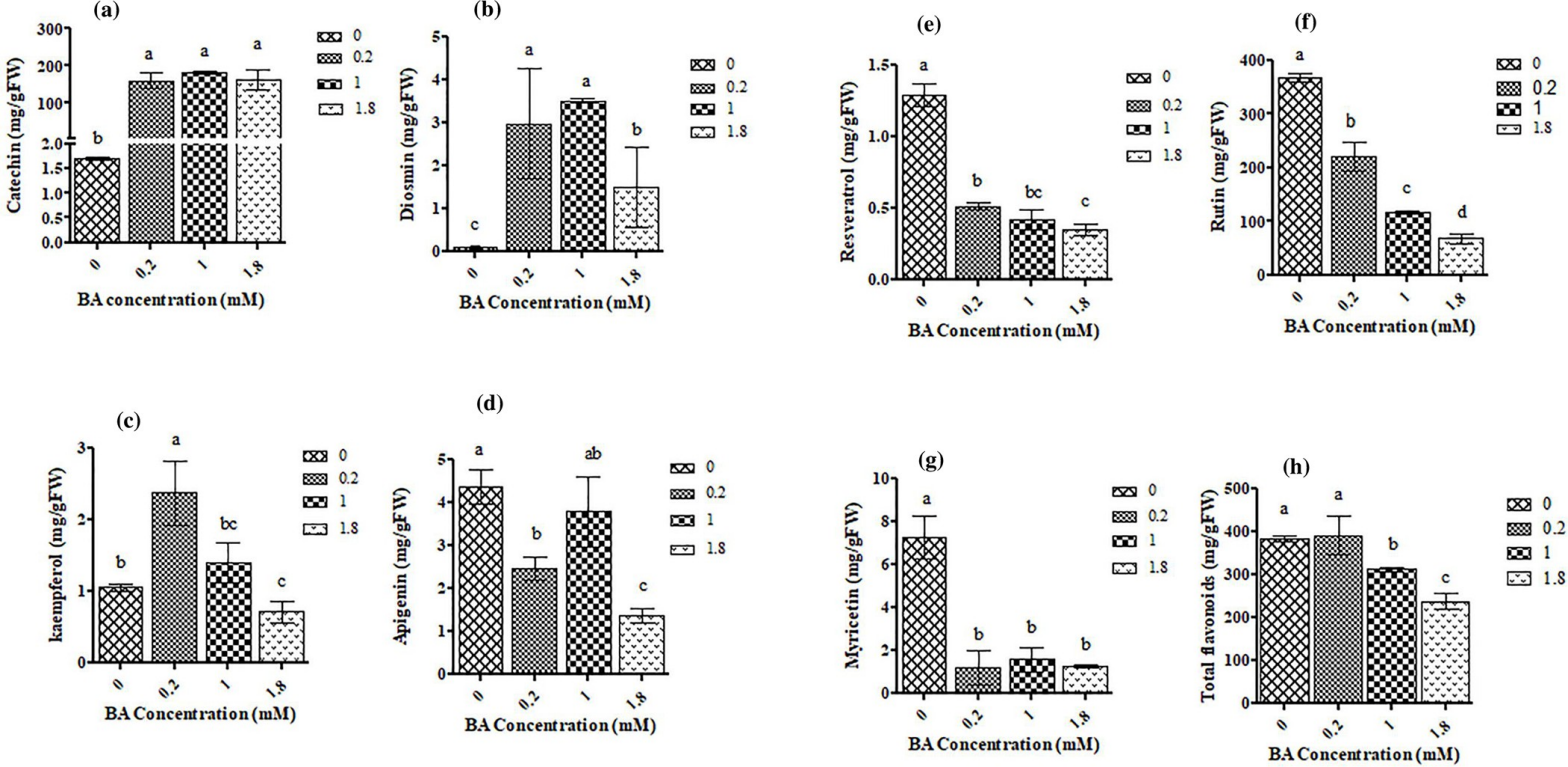
Alteration of *C*. *tarraconensis* flavonoids content in response to various concentrations of BA. Data are presented as mean ± SD, n = 3. Different letters show significant differences at *P* < 0.05 according to Duncan test.

Among flavonoids detected in *C*. *tarraconensis* catechin and diosmin of BA-treated samples were remarkably higher than controls (Fig 4A and 4B). Except for 0.2mM in other BA concentrations Kaempferol content was lower than control (Fig 4C). Apigenin content of *C*. *tarraconensis* at 1 mM BA showed no change, but reduced at other BA concentrations, compared with control (Fig 4D). The contents of myricetin, rutin, and stilbenoid resveratrol were reduced by all concentrations of BA, compared to controls (Fig 4E and 4G). Except for 0.2 mM of BA, the contents of total flavonoids were reduced in 1 and 1.8 mM compared to control (Fig 4H).

### Classification among different molecules with taxanes

**Fig 5:**
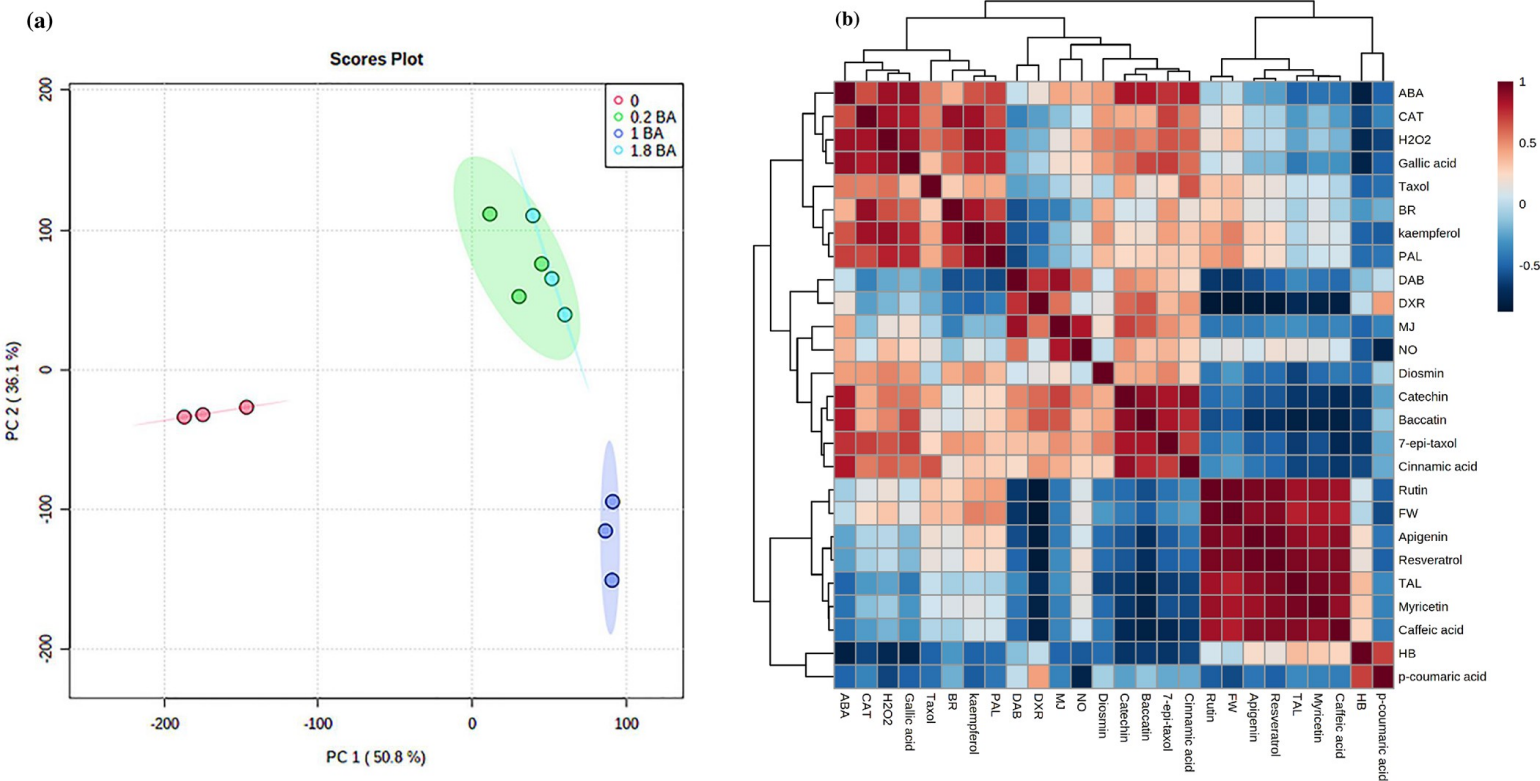
Correlation analysis of metabolites of *C*. *tarraconensis*, treated with BA showed by PCA. **(a)** and HCA **(b)**. Blue and red respectively show negative and positive correlations in HCA.

Based on PCA analysis, the total variance was accounted for 86.9% with 50.8% and 36.1% of the total variance for PC1 and PC2, respectively (Fig 5A). The 2 components showed clear segregation according to different concentrations of BA. High diversity was detected in 1mM of BA treatment indicating that this concentration significantly impressed the total variance in the collection of data (Fig 5A). Positive correlations between ABA and baccatin III, and between ABA and 7- epi Taxol were shown based on HCA analysis on taxanes and signal molecules (Fig 5B). Moreover, positive correlations were observed between MJ and DAB, between MJ and baccatin III, between BR and DAB, and between H2O2 with Paclitaxel and 7- epi Taxol (Table 2).

**Table 2:** The correlations between taxanes and signal molecules, enzymes, phenolic acids and flavonoids in *C*. *tarraconensis*.

	Taxanes			
**Signal Molecules**	10-Deacetyl baccatinIII	BaccatinIII	Paclitaxel	7-epi Taxol
H_2_O_2_	-	-	0.58	0.65
**ABA**	-	0.81	-	0.73
**MJ**	0.86	0.64	-	-
**BR**	0.57	-	-	-
**enzymes**				
**PAL**	-0.6	-	-	-
**TAL**	-	-0.8	-	-0.64
**DXR**	0.74	0.65	-	-
**CAT**	-	-	-	0.7
**phenolic acids**				
**Cinnamic acid**	-	0.77	0.65	0.71
**Gallic acid**	-	0.68	-	0.71
**Caffeic acid**	-	-0.77	-	-0.56
**HBA**	-	-0.64	-	0.66
**flavonoids**				
**Catechin**	-	0.9	-	0.82
**Myricetin**	-	-0.77	-	-0.56
**Resveratrol**	-	-0.7	-	-
**Apigenin**	-0.**6**	-0.7	-	-
**Rutin**	-0.66	-	-	-

HCA analysis on the correlations between taxanes and the enzymes which are involved in its biosynthesis showed negative correlations between PAL and DAB and also between TAL and baccatin III and 7-epi Taxol (Table 2). There were positive correlations between CAT and 7-epi Taxol, DXR and DAB and DXR with baccatin III (Table 2).

Among phenolic acids positive correlations were found between cinnamic acid with baccatin III, 7- epi Taxol, and Paclitaxel (Table 2). Positive correlations were also found between gallic acid with baccatin III and 7- epi Taxol (Table 2). Caffeic acid was negatively correlated with baccatin III and 7-epi Taxol (Table 2) likewise, HB was negatively correlated with baccatin III and 7-epi Taxol (Table 2).

Except for catechin which had a positive correlation with baccatin III and 7-epi Taxol (Table 2), other flavonoids were negatively correlated with taxanes. Negative correlations between myricetin and baccatin III, myricetin and 7-epi Taxol, resveratrol with baccatin III, apigenin with DAB, apigenin with baccatin III, rutin with DAB are some instances (Table 2).

**Fig 6:**
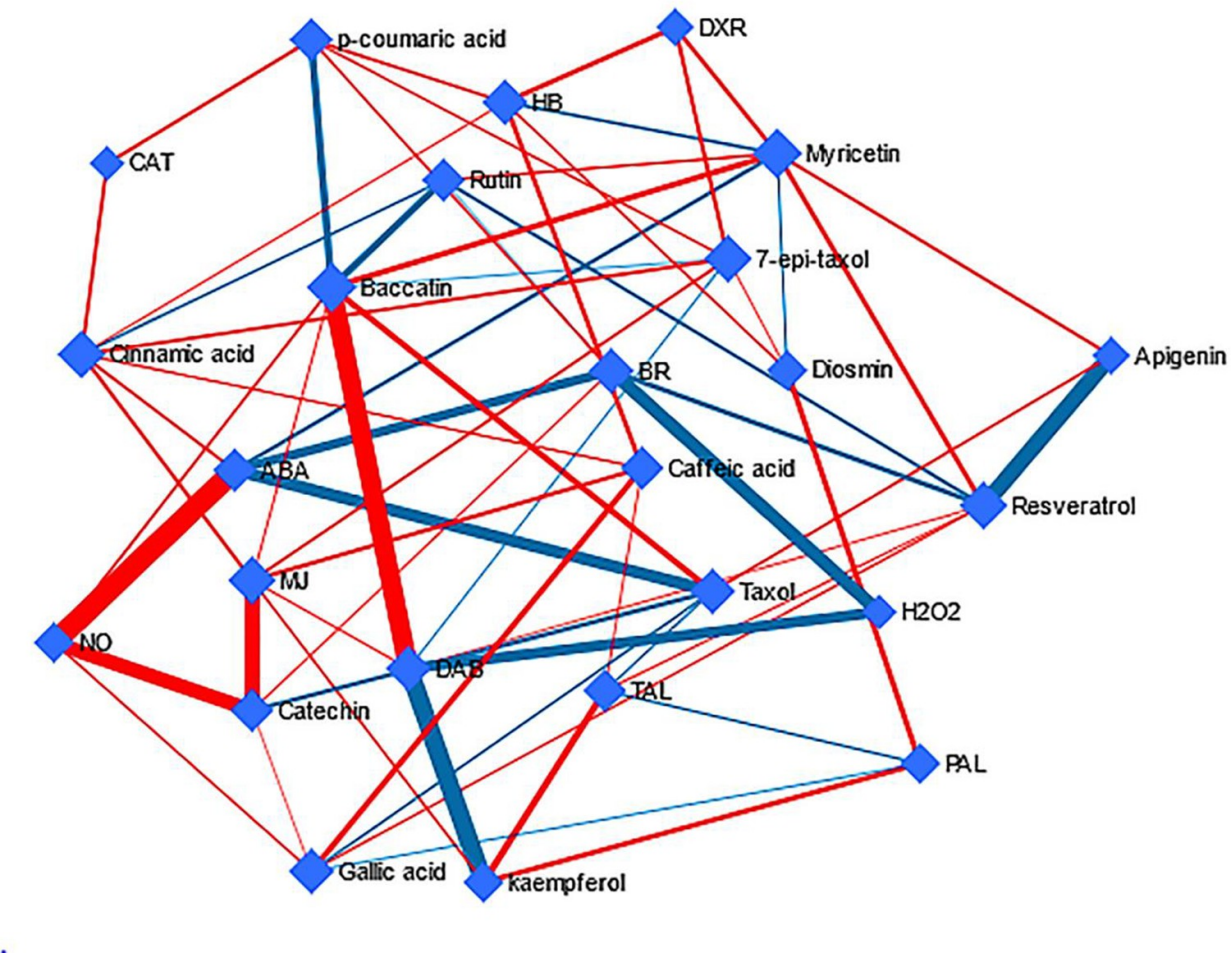
Network building between taxanes, phenolic acids and flavonoids based on DSPC analysis. Nodes demonstrate reconstructing correlation-based networks of compounds and the edge thickness is proportional to the strength of the shown correlation.

Based on DSPC network, various metabolites were detected with the nodes, while the associations between these metabolites were presented by lines (Fig 6). The data were normalized by the log or cubic root. In the network some of taxanes had central positions with different associations with other metabolites. Baccatin III with 8 correlation edges, 7 epi taxol with 7 correlation edges and DAB and Paclitaxel with 6 nodes are some instances. Paclitaxel was positively associated with baccatin and apigenin, while negatively associated with ABA, catechin, gallic acid and TAL. Moreover, in order to achieve comprehensive information about the most disturbed pathways and differential metabolites induced by BA, metabolic pathway network was constructed (Fig 6).

## Discussion

The biosynthesis pathway of Paclitaxel needs a large number of precursors, intermediates, and derivatives whose interactions contribute to the variations in the amounts of Paclitaxel [10]. Treatment of *C*. *tarraconensis* with different concentrations of BA promoted variations in signal molecules such as H_2_O_2_ and triggered a cascade of downstream events resulting in a shift of carbon flux instead of biomass production toward the production of secondary metabolites e.g., phenylpropanoids and taxanes. Treatment of *C*. *tarraconensis* with 0.2 mM BA was accompanied by the drastic increase in H_2_O_2_ and ABA. The production of both ABA and H_2_O_2_ as signaling molecules are intertwined. It is evidenced by the presence of both G-box (ABA responsive-) and antioxidant responsive elements in the promoter of the CAT gene [21]. Interestingly, an increase of ABA and H_2_O_2_ in 0.2 mM BA-treated *C*. *tarraconensis* was associated with the increase of CAT activity. CAT functions as the main antioxidant enzyme in eradication of produced H_2_O_2_ in stress condition [22].

Another bioactive signal molecule is NO whose connection the different metabolites has been demonstrated. It has been shown that ameliorative impact of NO in plants under abiotic stress conditions is closely related to reduction of ROS [23, 24]. Application of NO scavenger and inhibitors of its producing enzymes resulted in reduction of anti-oxidant enzymes activities in maize, emphasizing the role of NO in upregulation of subcellular anti-oxidant enzymes [25].

In agreement with their findings, in the present study decomposition of H_2_O_2_ at 1- and 1.8-mM BA-treated *C*. *tarraconensis* was accompanied by the increase of NO.

Role of NO in activating another signaling molecule MJ has been already documented [26]. The existence of cross-talk between NO and MJ signaling was observed in *Arabidopsis* under wounding stress where NO activated early MJ signaling genes, and MJ triggered feedback of NO accumulation [27]. On the other hand, MJ stimulates NO production in *Taxus* cells and motivated defense responses, suggesting that MJ is upstream of NO [28]. Similar tendencies were observed in the increase of NO and MJ in *C*. *tarraconensis* after treatment with 1- and 1.8-mM BA.

MJ induces diterpenoids by influencing DXR gene and Producing geranylgeranyl diphosphate, DXR is a key enzyme in the early steps of diterpenes and diterpene alkaloids e.g., Paclitaxel. Exogenous application of MJ caused a ca. 2 fold increase in DXR gene expression and diterpenoid tanshinone in *Salvia castanea* hairy roots [29]. In cultures hazel cells exposure to low frequency ultrasound increase the gene expression and activity of DXR to 2 and 4fold of the control which was accompanied by 6 fold increase of Paclitaxel [20]. In the present study increase of MJ in an BA-treated *C*. *tarraconensis* resulted in outstanding increase of DXR activity and remarkable increase of taxanes production, to a large extent DAB and baccatin III and to a less extent Paclitaxel.

Paclitaxel is composed of a taxan ring which is synthesized through terpenoid pathway and a C-13 side chain which is derived from phenylalanine via phenylalanine ammonia mutase (PAM). The latter catalyzes a rate-limiting step for Taxol production [7]. Similarity searches indicate that PAM is very closely related to PAL, a key enzyme in the phenylpropanoid biosynthesis pathway. However, the Km for PAL is much lower than PAM, suggesting that if the phenylpropanoid and the taxoid pathways were in direct competition for substrate then the phenylpropanoid pathway would be favored [7].

In comparison, Paclitaxel content of *C*. *tarraconensis* increased up to twice of the control, while cinnamic acid (the product of PAL) increased up to15 fold of controls in BA-treated samples.

It has been proved that *trans*-cinnamic acid reduces the activity of PAL by 40–50% [30], while reducing paclitaxel accumulation by 90% [31]. That is why in higher concentrations of BA, overproduction of cinnamic acid in *C*. *tarraconensis* not only reduced the activity of PAL but also decreased Paclitaxel content to the control level. DSPC algorithm derived network confirmed that most compounds with close proximity have shared metabolic pathway relationships.

In particular, cinnamic acid impressed on paclitaxel and its derivatives, but also on the accumulation of Paclitaxel precursors.

Negative correlations were observed between contents of toxoids and flavonoids of *C*. *tarraconensis* after treatment with BA. A negative correlation was also reported between Paclitaxel and flavonoid biosynthesis in hazel cells after treatment with salicylic acid and ultrasound [32]. In plants this has been attributed to the opposite effects of certain helix-loop-helix transcription factors which positively regulates flavonoid biosynthesis genes meanwhile negatively controls the expression of genes involved in taxanes biosynthesis pathway [33]. Whether or not such associations exist between Paclitaxel and flavonoids in Paclitaxel producing endophyte fungi, needs to be clarified by further investigations.

In conclusion, our findings revealed that profiles of several metabolites significantly altered in response to BA treatment. Although Paclitaxel biosynthesis is initially stated with terpenoids biosynthesis pathway, PAL and phenylpropanoids have an undeniable role in biosynthesis of C13 side chain and accumulation of Paclitaxel in fungi.
